# Evolving Trends in Organ Donation and Transplantation Rates Across Muslim Majority Countries

**DOI:** 10.3389/ti.2025.15116

**Published:** 2025-11-24

**Authors:** Fatima Malik, Mehreen Khan Bhettani, Junaid Mansoor, Zainab Arslan, Muhammad Shamim Khan, Irum Amin, Shahid Farid, Usman Haroon, Zubir Ahmed, Muhammad Khurram, Rhana Zakri, Adnan Sharif

**Affiliations:** 1 Guy’s & St Thomas’ Hospital, London, United Kingdom; 2 International Pakistani Association of Transplantation (IPAT), London, United Kingdom; 3 Great Ormond Street Hospital for Children, London, United Kingdom; 4 UCL Great Ormond Street Institute of Child Health, London, United Kingdom; 5 Cambridge University Hospitals, Cambridge, United Kingdom; 6 St James’s University Hospital, Leeds, United Kingdom; 7 Beaumont Hospital, Dublin, Ireland; 8 Queen Elizabeth University Hospital, Glasgow, United Kingdom; 9 Royal London Hospital, London, United Kingdom; 10 Queen Mary University of London, London, United Kingdom; 11 University Hospital Birmingham NHS Foundation Trust, Birmingham, United Kingdom; 12 School of Infection, Inflammation and Immunology, University of Birmingham, Birmingham, United Kingdom

**Keywords:** Islam, Muslims, organ donation, transplantation, resource

## Abstract

Muslim-majority countries differ in socio-cultural behavior and economic development but share a similar high burden of organ failure. Due to this heterogeneity, mapping organ donation and transplantation activity is of interest for future healthcare provision. Data was analyzed for 50 Muslim-majority countries (defined as Muslims comprising >50% of the population). Organ donation/transplantation rates were obtained from global registries between 2013–2023. Supplementary socio-economic and health data were obtained from open-source data repositories. Muslim-majority countries population increased from 1.53 billion to 1.88 billion between 2013–2023. Organ donation/transplant activity was only reported for 21/50 countries. Most organ donations came from living people rather than deceased donors (resulting in kidney and liver transplantation being the most common procedures). Other transplant activity rates were low. Poisson regression analyses identified multiple socioeconomic indicators to be associated with deceased- or living-donor activity, while negative binomial analyses comparing Muslim-majority to other countries within the region showed Muslim countries had lower deceased donation rates. Our study shows access to transplantation is lacking in many Muslim-majority countries. While socio-economic factors play a role, other challenges like religious and/or cultural barriers must be appreciated. With such global heterogeneity, bespoke country-specific interventions are warranted to improve transplantation opportunities in Muslim-majority countries.

## Graphical Abstract

**Figure:**
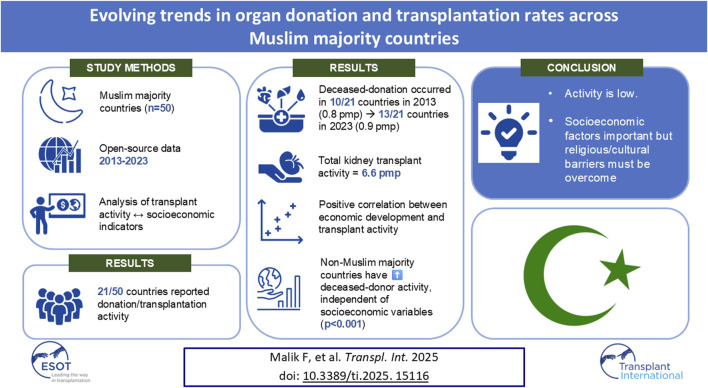

## Introduction

Organ donation and transplantation are critical components of modern healthcare systems, offering life-enhancing or life-saving solutions to people suffering from end-stage organ failure. However, accessibility to transplantation for organ failure patients is not ubiquitous across the world [1]. There is significant heterogeneity in observed rates of organ donation and transplantation both within [2] and between countries [1], shaped by multi-factorial variables that include (but are not limited to) socio-cultural influences, resource constraints, necessary infrastructure and economic development. Such inequity leads to major health disparities and sub-optimal survival outcomes for people living with end-stage organ failure across the globe.

In Muslim-majority countries, defined as those where Islam is the predominant religion and plays a central role in shaping societal norms, the approach to organ donation is particularly complex. While there is increasing acceptance of the merits of organ donation to facilitate transplantation in many Muslim-majority countries, with theological and religious scholarly rulings in support, there remains a diverse range of practices and beliefs [3]. In parallel, despite religious unity, Muslim-majority countries represent a diverse and heterogenous group of countries scattered across the globe with varying degrees of socio-economic development and cultural legacies. Many Muslim-majority countries are recognized as low or middle-income countries (LMIC), and the burden of end-stage kidney disease [4], liver disease [5], heart failure [6] and lung failure [7] in such countries is well described. The requirement for transplantation is likely to be high in these countries and improving equity of access is acknowledged as an important policy innovation for countries [1]. However, developing targeted policy innovations requires an understanding of the current landscape and an exploration of any inter-country variation that probes heterogenous activity data.

Despite a high requirement for solid organ transplantation due to the underlying burden of end-stage organ disease, it is unclear what level of organ donation and transplantation activity exists in Muslim-majority countries. A previous narrative review reported some granular data regarding organ donation models across the 57 member states of The Organization of Islamic Cooperation, with limited analyses to explore the data further [8]. This is important to understand, as efforts to mitigate disparity of access must be undertaken in the context of current landscape realities. Distinguishing religious obstacles from socio-economic capacity as barriers to facilitate organ donation and transplantation infrastructure is critical. To date, no study has explored the evolving trends of organ donation and transplantation activity across Muslim-majority countries or studied variables that may impact upon such activity. As a sizable population cohort, understanding the scale of organ donation and transplantation activity among Muslim majority countries is important from a global healthcare perspective.

In this article, we aim to explore evolving trends in organ donation and transplantation across Muslim-majority countries over the last decade, providing a snapshot of activity across these heterogenous countries. By analyzing organ donation and transplant rates alongside key socio-economic indicators, this study aims to understand how Muslim-majority countries may successfully navigate organ donation and transplantation challenges.

## Materials and Methods

### Country Selection

For this analysis, Muslim-majority countries were identified based on the proportion of the national population practicing Islam. For inclusion in this study, we selected those countries where more than 50% of the population is identified as Muslim, based on the most recent data available from the Pew Research Centre or the United Nations Demographic and Social Statistics report (see data sources and links below). This threshold ensured that any selected country possessed a predominantly Islamic cultural and social context, essential for examining organ donation and transplantation activity influenced by religious or societal norms. Countries were excluded if current data on religious composition was unavailable or deemed unreliable.

### Data Sources

This study relied exclusively on freely available and publicly accessible data sources to assess organ donation and transplantation activity in Muslim-majority countries. Country profile information (including religious composition) was obtained from the Pew Research Centre [9] and the United Nations Demographic and Social Statistics report [10].

Organ donation and transplantation rates were obtained from the Global Observatory for Donation and Transplantation (GODT) for the latest available year (2023 in most cases) [11]. We referred to the International Registry on Organ Donation and Transplantation (IRoDaT) [12] if relevant data did not exist in the GODT.

Supplementary socio-economic and health data were obtained from data repositories including World Health Organization (WHO) [13], International Monetary Fund (IMF) [14] and the World Bank [15]. Chronic kidney disease (CKD) data was obtained from the Global Burden of Disease Study 2021 [16].

### Variables of Interest

GODT and IRoDaT data were used to obtain deceased and living donor activity rates with corresponding solid organ transplantation activity data. Organ donation and transplantation activity was reported per million population (pmp). Other data sources were used for collating socio-economic variables which included population (in millions), Gross Domestic Product (GDP; *per capita* and per person), health expenditure (% of GDP), road traffic accidents (RTA) per 100,000 population, literacy rate among adults (%), life expectancy (in years), unemployment rate (%) and world economy rank.

### Statistical Analysis

For categorical variables, chi-squared tests were employed to assess associations between different categories, such as country-specific donation rates or organ transplantation activity. For continuous variables, the Mann-Whitney U test was utilized to compare distributions, especially in cases where the data were not normally distributed or the sample sizes were unequal. To gain a comprehensive understanding of the data, several descriptive parameters were calculated, including the mean, median, standard deviation, and range, which provided a detailed overview of the central tendencies and variability of the variables in question.

Additionally, we performed multivariable Poisson regression or, for data with overdispersion, a negative binomial analysis to explore independent variables such as socio-economic factors and/or Muslim-majority status that may potentially confound the outcome variable (e.g., deceased or living donor rates). For the latter, we restricted the analyses to specific regions based upon World Bank classification that represented a variety of countries but encompassed most Muslim-majority countries. These regions were South Asia, Central Asia, Middle East, North Africa, Sub-Saharan Africa and East Asia, which included 47/50 Muslim-majority countries. Poisson or negative binomial results were reported as incidence rate ratios (IRR) with 95% confidence intervals (CI). All statistical analyses were carried out using R version 4.4.2.

## Results

### Country Profiles

**FIGURE 1:**
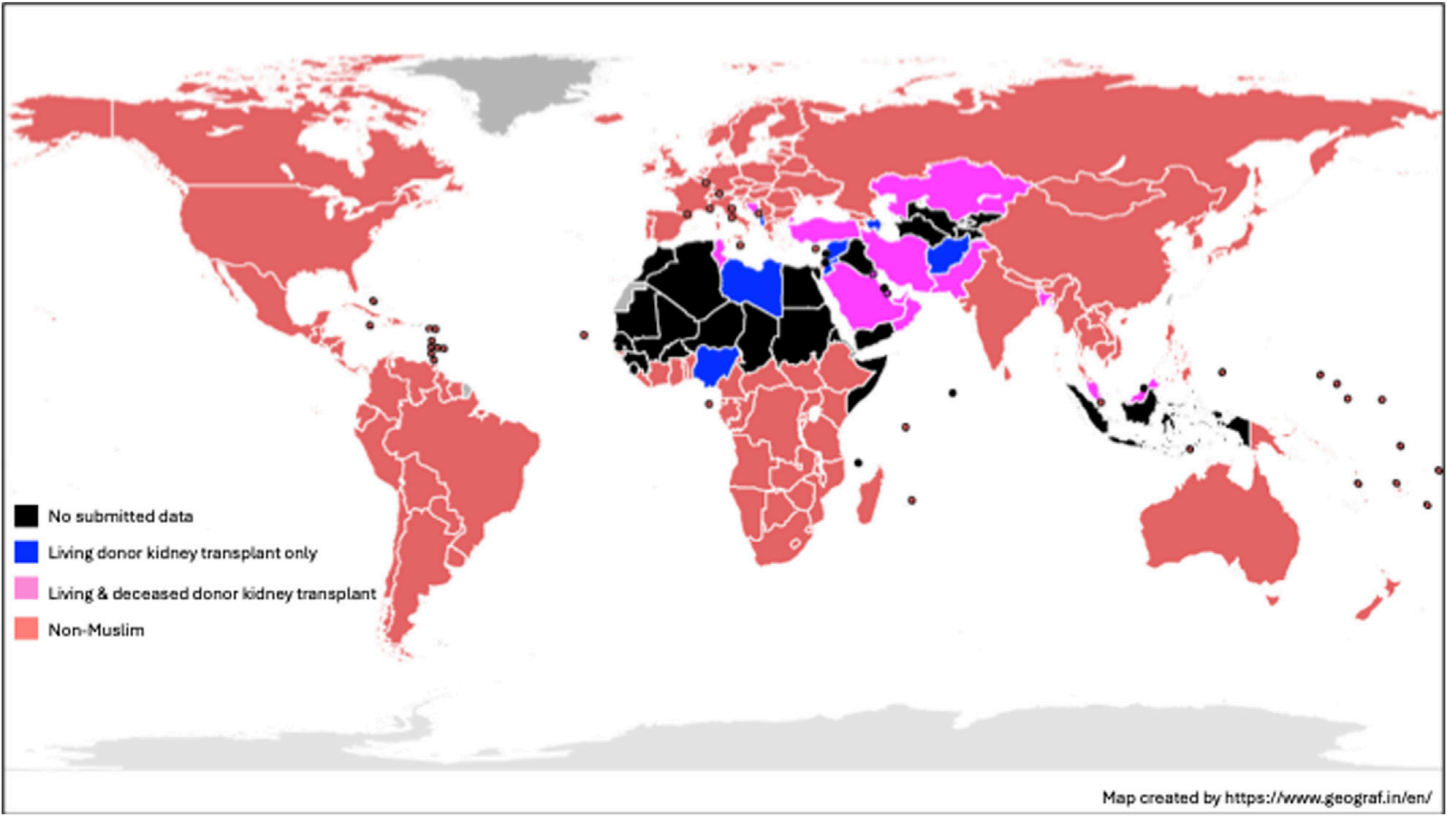
World map of Muslim-majority countries and kidney transplant activity based upon 2023 data.

In total, 50 countries were identified as having a Muslim majority with 47 located in Asia or Africa (see Figure 1). Data completeness was excellent for socio-economic variables; population, GDP, life expectancy, world economic rank (100.0%), health expenditure (93.9%), literacy rate (93.9%), unemployment rate (98.0%) and RTA mortality rate (98.0%). However, there was significant degree of missingness in the organ donation and transplantation activity data, with a range from 38.8% (total kidney transplant activity) to 30.6% (simultaneous pancreas-kidney kidney transplant activity).

**FIGURE 2:**
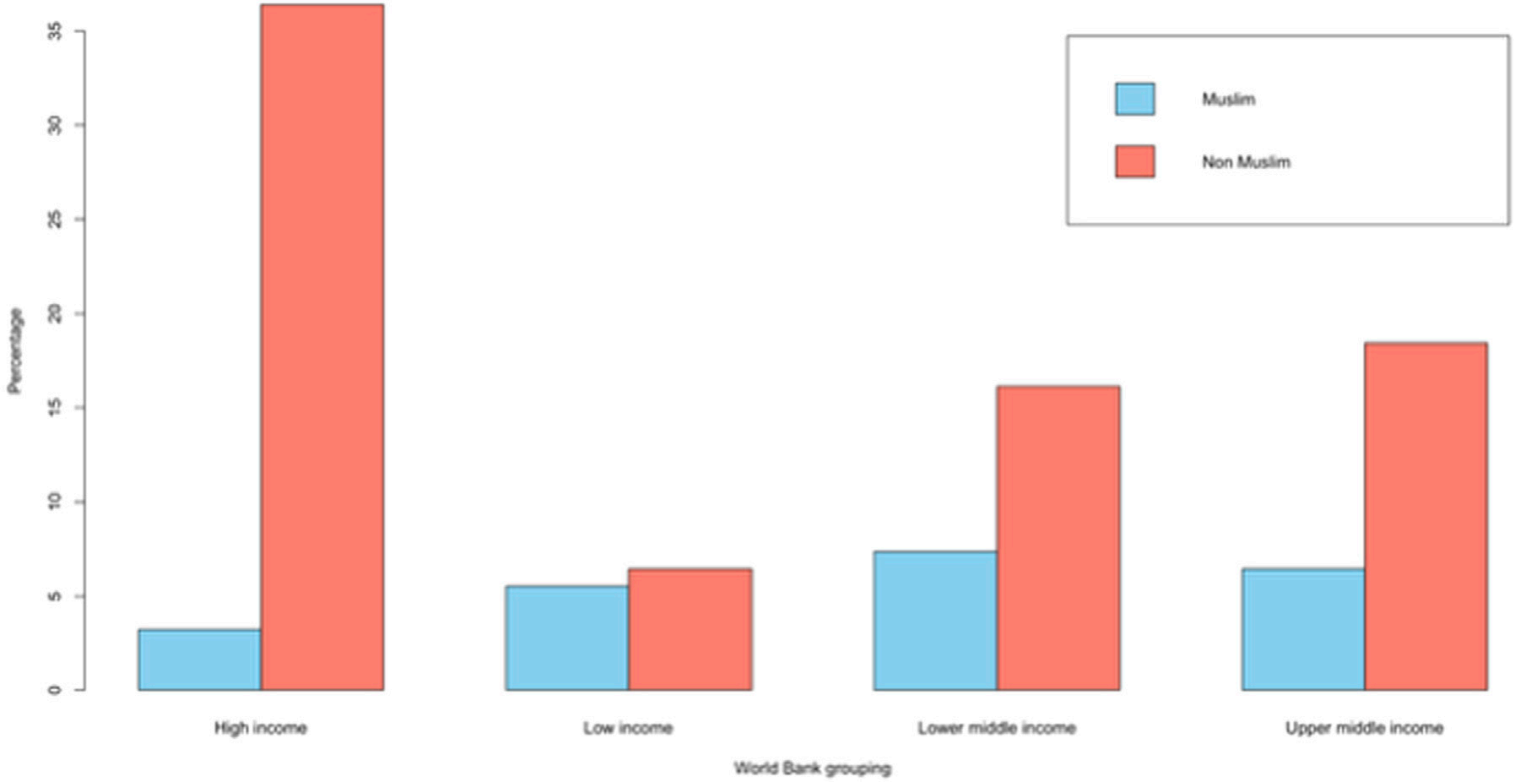
Living and deceased donor activity across Muslim-majority countries in 2023.

The global Muslim population increased from 1.53 billion to 1.88 billion between 2013 and 2023 (representing a 22.9% increase over the decade). With an estimated global population of 8 billion people in 2023 according to the United Nations [17], this represents 23.5% of the world population. A summary of individual country profiles is shown in Table 1. From the 50 Muslim-majority countries, 49 were categorized by their World Development Indicator defined by the World Bank (no Palestinian data recorded). Compared to non-Muslim majority countries, Muslim-majority countries were disproportionately more likely to be classed in a category other than high-income (see Figure 2).

**TABLE 1:** Muslim-majority country profiles.

Country	Population in 2023 (m)	GDP (*per capita*)	GDP (*per capita*) PPP	Health expenditure (% of GDP)	RTA (mortality per 100,000 population)	Literacy rate, adult total (%)	Life expectancy (years)	Unemployment total (%)	World economy rank
Afghanistan	42.2	352.6	2,173	21.83	24.1	37	63	14.4	111
Albania	2.7	8,367.80	20,018	7.27	10.8	99	77	11.6	124
Algeria	45.6	5,260.20	16,900	5.53	18.3	81	77	11.8	40
Azerbaijan	10.1	7,155.10	23,660	4.7	17.2	100	73	5.6	71
Bahrain	1.5	29,084.30	63,497	4.27	8.1	98	79	1.2	99
Bangladesh	173.0	2,529.10	9,211	2.36	18.6	76	74	5.1	24
Bosnia and Herzegovina	3.2	8,426.10	20,431	9.56	13.7	98	75	10.4	110
Brunei Darussalam	0.5	33,430.90	86,866	2.2	3.6	98	75	5.3	137
Burkina Faso	23.3	874.1	2,712	6.38	27.8	34	60	5.3	114
Chad	18.3	719.4	2,757	5.19	26.4	27	53	1.1	131
Comoros	0.9	1,587.20	3,725	6.34	29	62	64	5.8	179
Djibouti	1.1	3,606.40	7,988	2.88	23.3	No data	63	26.3	165
Egypt	112.7	3,512.60	20,180	4.61	9.4	75	70	7.3	17
Eritrea	3.7	643.8	712	4.15	17.7	77	67	5.9	171
Gambia	2.8	843.8	3,318	3.19	22	59	63	6.5	164
Guinea	14.2	1,663.90	4,156	3.76	37.4	45	59	5.3	117
Indonesia	277.5	4,940.50	15,553	3.71	11.3	96	68	3.4	8
Iran (Islamic Republic of)	89.2	4,502.50	18,658	5.77	20.6	89	75	9.1	23
Iraq	45.5	5,512.50	14,766	5.25	21.5	86	71	15.5	46
Jordan	11.3	4,482.10	No data	7.29	13.6	95	74	17.9	94
Kazakhstan	19.9	13,136.60	39,463	3.92	12.2	100	74	4.8	39
Kosovo	1.8	5,943.10	15,864	No data	No data	No data	80	No data	153
Kuwait	4.3	37,533.20	50,933	5.78	9.2	96	80	2.1	72
Kyrgyzstan	7.1	1,969.90	7,279	5.44	13.3	100	72	4	128
Lebanon	5.4	3,823.90	12,453	10.06	9.7	93	74	11.6	108
Libyan Arab Jamahiriya	6.9	7,330.00	14,781	4.02	34	No data	72	18.7	98
Malaysia	34.3	11,648.70	38,693	4.38	13.9	96	76	3.9	29
Maldives	0.5	12,667.40	32,541	10.03	1.3	98	81	4.1	158
Mali	23.3	897.4	2,762	4.47	20.2	31	59	3	115
Mauritania	4.9	2,149.40	7,874	4.12	9.5	67	65	10.5	144
Morocco	37.8	3,672.10	10,180	5.74	18.6	77	75	9.1	57
Niger	27.2	618.3	1,824	5.81	24.9	38	62	0.6	126
Nigeria	223.8	1,621.10	6,366	4.08	17.2	63	54	3.1	27
Oman	4.6	23,295.30	41,558	4.37	11	97	74	1.5	77
Pakistan	240.5	1,407.00	6,530	2.91	11.9	58	66	5.5	26
Palestine	5.2	3,367.6	3,372	No data	5	98	73	24.4	148
Qatar	2.7	87,480.40	111,789	2.89	7.3	98	82	0.1	61
Saudi Arabia	36.9	28,895.00	61,932	5.97	18.5	98	78	4.9	18
Senegal	17.8	1,746.00	4,786	4.35	20.8	58	68	2.9	102
Sierra Leone	8.8	433.4	3,359	8.55	13.8	49	60	3.2	151
Somalia	18.1	643.8	1,780		20.2	54	56	19	150
Sudan	48.1	2,272.50	3,158	2.84	19.6	61	66	11.4	95
Syrian Arab Republic	23.2	421.1	753	3.05	29.9	94	72	13.5	92
Tajikistan	10.1	1,189.00	5,147	8.01	13.9	100	71	7	125
Tunisia	12.5	3,895.40	13,892	6.97	16.3	85	74	15.1	83
Türkiye	85.3	12,985.80	38,390	4.57	6.5	97	78	9.4	12
Turkmenistan	6.5	9,190.70	No data	5.57	8	99	69	4.1	82
United Arab Emirates	9.5	52,976.80	74,713	5.31	5.9	98	79	2.7	38
Uzbekistan	36.4	2,496.10	10,992	7.74	9.3	100	72	4.5	56
Yemen	34.4	701.7	2,012	4.25	29.8	54	64	17.2	112

### Organ Donation and Transplantation Activity

**FIGURE 3:**
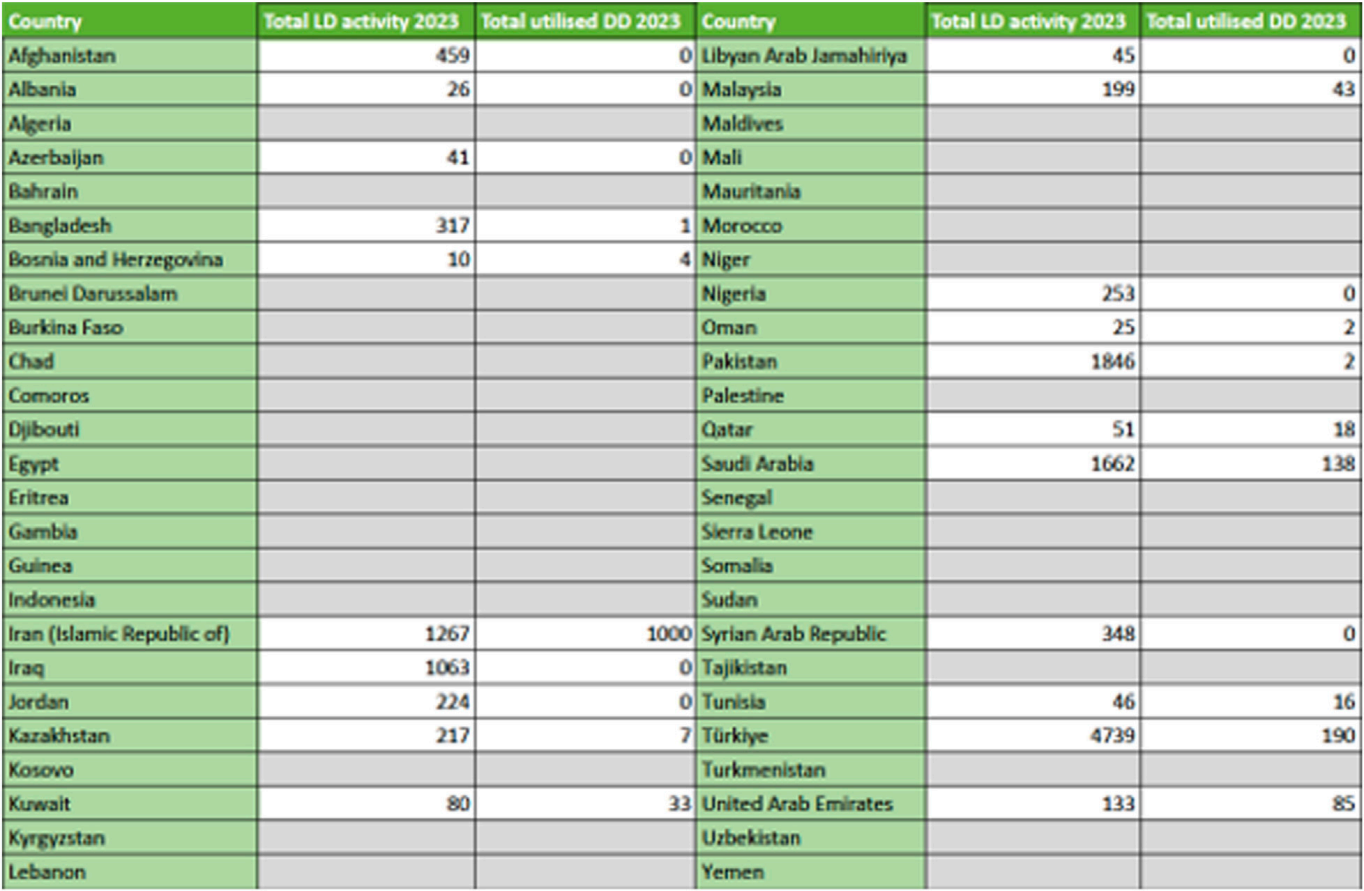
World Bank defined income category status comparing Muslim-majority versus other countries.

Organ donation and transplant activity data was reported from 21 countries, but none from 29 countries. Data for the latest available year is summarized in Figure 3. Total organ donor activity increased from 12,557 in 2013 (8.2 pmp) to 15,950 in 2023 (8.5 pmp). Deceased donation occurred in 10/21 and 13/21 countries in 2013 and 2023 respectively. Total actual deceased donors were 1,173 in 2013 (0.8 pmp) and 1,681 in 2023 (0.9 pmp), representing a 43.3% increase over the decade but only a marginal increase per million population in the context of population growth. This increase was almost exclusively in the context of donation after brain death (DBD), with only one country (Bosnia and Herzegovina) reporting donation after circulatory death (DCD) activity. A larger rise was seen in living donor activity. For example, living kidney transplant activity increased by 17.1% over the decade from 8,841 in 2013 (representing 82.0% of total kidney transplant activity at that time, n = 10,781) to 10,356 in 2023 (representing 83.2% of total kidney transplant activity at that time, n = 12,440). However, per million population this represents 5.8 pmp and 5.5 pmp living donor rates in 2013 and 2023 respectively, indicating stagnant rates despite population growth. Liver transplant activity increased by 53.9% from 2,543 in 2013 (65.6% of activity being derived from living donors) to 3,913 in 2023 (68.9% of activity being derived from living donors), equating to 1.7 pmp and 2.1 pmp respectively.

The observed rise in deceased donor rates only led to a marginal 7.4% increase in actual deceased donor kidney transplant rates from 1,940 in 2013 to 2,084 in 2023 (1.3 pmp and 1.1 pmp respectively). There was a higher 33.9% increase in actual deceased donor liver transplant rates from 901 in 2013 to 1,206 in 2023 (0.6 pmp and 0.6 pmp respectively). Transplant rates using other solid organs were low, reflective of such activity being derived from deceased donors only. Rates for heart/lung transplantation in 2013 and 2023 were 243 (0.2 pmp) and 329 (0.2 pmp) respectively (reported in 7 countries only), with a smaller number of pancreas transplants in 2013 (n = 37, <0.1 pmp)) and even lower numbers (n = 24, <0.1 pmp) in 2023 having been reported from 3 to 5 countries respectively). The lowest rates were reported for small bowel transplantation; just 3 countries performing a total of 14 in 2013 and 13 in 2023 (both <0.1 pmp). For the latest year available (relying upon registry data records only), countries that reported any non-kidney or liver-related transplant activity were as follows: heart/lung transplantation (Iran, Kazakhstan, Kuwait, Saudi Arabia, Tunisia, Turkey and the United Arab Emirates), pancreas transplantation (Iran, Kuwait, Saudi Arabia, Turkey and the United Arab Emirates) and small bowel transplantation (Iran, Saudi Arabia and Turkey).

### Burden of Kidney Failure and Kidney Transplantation Activity

As kidney transplantation is the commonest solid organ transplant procedure, with the best cost effectiveness argument in the context of end-stage kidney failure, we explored the burden of chronic and end-stage kidney disease and its association with countries profile. Reviewing raw data from the Global Burden of Disease study 2021 [16], prevalence and death rates from chronic kidney disease were 134,048,251 and 207,102 respectively across Muslim-majority countries (see Supplementary Table S1). For the latest year available, kidney transplantation activity data were reported for 21 out of 50 Muslim-majority countries as highlighted above. Total kidney transplant activity was 12,440 at 6.6 pmp (deceased and living donor transplantation activity was 2,084 and 10,356 respectively). Only 13 countries reported both deceased and living donor kidney transplant activity, with 8 exclusively using living kidney donors only.

### Adjusted Regression Analyses Comparing Muslim-Majority Countries

We performed a multivariable Poisson regression analysis to explore the impact of socio-economic variables on deceased or living donor activity (see Table 2). Socio-economic factors were significantly associated with IRRs, with some intuitive findings such as increased activity rates for both deceased- and living-donor activity with every unit increase in health expenditure or improved world economy ranking. However, some differences were also observed. For example, every unit increase in literacy rates was associated with lower deceased donor but higher living donor activity. In contrast, every unit increase in road traffic accidents was associated with higher deceased donor but lower living donor activity.

**TABLE 2:** Poisson regression exploring socio-economic indicators and deceased or living donor activity among Muslim-majority countries.

Variable	Deceased donor activity			Living donor activity		
	IRR	95% CI	p value	IRR	95% CI	p value
Population in 2023	1.000	1.000–1.000	<0.001	1.000	1.000–1.000	<0.001
Gross domestic product (*per capita*) PPP	0.999	0.999–0.999	<0.001	1.000	1.000–1.000	<0.001
Health expenditure (% of GDP)	1.560	1.435–1.694	<0.001	1.401	1.356–1.446	<0.001
Literacy rate (% adult total)	0.922	0.883–0.959	<0.001	1.073	1.061–1.086	<0.001
Unemployment rate (%)	0.999	0.961–1.039	0.954	1.231	1.209–1.254	<0.001
Road traffic accidents (mortality per 100,000)	1.109	1.093–1.126	<0.001	0.999	0.993–1.005	0.766
Life expectancy	2.656	2.366–3.000	<0.001	0.887	0.867–0.906	<0.001
World economy rank	0.910	0.900–0.920	<0.001	0.960	0.957–0.963	<0.001

### Adjusted Regression Analyses Comparing Muslim-Majority Countries

Due to overdispersion of the data, we performed a negative binomial analysis to explore the impact of socio-economic variables in addition to Muslim-majority status on deceased or living donor activity. As seen in Table 3, non-Muslim-majority countries were significantly more likely to have higher deceased donor activity but there was no difference in living donor activity. This observation was independent of socio-economic variables.

**TABLE 3:** Negative binomial analysis exploring socio-economic indicators and deceased or living donor activity among Muslim-majority countries.

Variable	Deceased donor activity			Living donor activity		
	IRR	95% CI	p value	IRR	95% CI	p value
Non-Muslim majority	4.058	2.989–5.521	<0.001	1.946	0.787–4.645	0.125
Population in 2023	1.000	1.000–1.000	<0.001	1.000	1.000–1.000	0.006
Gross domestic product (*per capita*) PPP	0.999	0.999–1.000	0.981	1.000	0.999–1.000	0.329
Health expenditure (% of GDP)	1.589	1.475–1.709	<0.001	1.695	1.353–2.097	<0.001
Literacy rate (% adult total)	1.024	0.997–1.051	0.071	1.087	0.962–1.219	0.110
Unemployment rate (%)	0.891	0.858–0.925	<0.001	1.060	0.912–1.233	0.409
Road traffic accidents (mortality per 100,000)	0.965	0.948–0.982	<0.001	0.912	0.860–0.968	0.001
Life expectancy	1.473	1.380–1.580	<0.001	0.968	0.880–1.068	0.499
World economy rank	0.949	0.942–0.955	<0.001	0.958	0.946–0.971	<0.001

## Discussion

In this study, we highlight the evolving trends in organ donation and transplantation activity across Muslim-majority countries and identify significant heterogeneity in access to transplantation. Most countries report no data and can be assumed to lack any regulated organ donation or transplantation activity. The remaining countries mostly have living donor activity but there has been little improvement in deceased donor activity over the last decade (which is almost exclusively donation after brain death only). There was an association between markers of advanced economic development (e.g., increased economic status, literacy rates and life expectancy) and transplantation activity. Within regions, Muslim-majority countries had less deceased donor activity but similar living donor activity independent of socio-economic factors. This data confirms that a significant gap exists in organ donation provision and access to transplantation services across the Muslim-majority world. Even among economically advanced Muslim-majority countries, organ donation activity rates (especially deceased donation) are sub-optimal in comparison to regional countries. Our data suggests religious and/or cultural barriers are likely to be as important as economic development to boost organ donation and meet transplantation needs in Muslim-majority countries.

Previous work in this area has either reported narrative summaries without further investigation [8] or explored the issue from a regional perspective only [18, 19]. It is well documented that organ donation rates among Muslim-majority countries are sub-optimal in comparison to countries with established organ donation and transplantation programs. When compared to countries like the United Kingdom (1,513 and 960 deceased and living donors respectively), Spain (2,346 and 437 deceased and living donors respectively) and the United States of America (16,336 and 6,942 deceased and living donors respectively) [11], it is sobering to observe low organ donation activity across Muslim-majority countries for the latest GODT data available. For example, the population of the United States according to the GODT registry for 2023 was 340 million persons; therefore, despite a fifth of the Muslim majority country population at 1.88 billion, the United States of America was achieving nearly 50% more organ donor activity. Barriers to develop organ donation and transplantation activity across Muslim-majority countries relate to appropriate staffing, resources and infrastructure. This is well known to the transplantation community [20]. However, there are important socio-cultural barriers that must be acknowledged specific to Muslim-majority countries. The commonly cited barrier of religious ambiguity may be resolved within the context of Islamic jurisprudence but swaying public opinion remains a challenge. While arguments for and against the use of organ donors (especially donation after brain death) within Islam are well versed [3], an overwhelming body of Muslim scholarly opinion agrees with all forms of organ donation being compatible with Islamic belief. However, public opinion does not automatically follow adopted scholarly opinion. Even among Western Muslims living in Muslim-minority countries with established organ donation and transplantation infrastructure, attitudes to organ donation can be ambivalent with significant reservations about religious barriers despite supportive religious scholarly opinion [21]. Promising results from a cluster, randomized-controlled trial using mosque-based, religiously tailored, ethically balanced education demonstrated significant kidney donation-related knowledge gains among Muslims in the United States [22]. Whether such interventions will help in Muslim-majority countries remains to be seen and there are likely to be varying religious and cultural factors that influence organ donation practice. However, while resolving religious and cultural issues to engage public opinion is critically important to address [23], this must be tackled in parallel to the immediate challenge of creating an appropriate infrastructure to support the establishment of a national organ donor procurement, allocation and transplantation service.

It is important to note the GODT and IRoDaT registries do not fully capture all organ donation and transplantation activity. For example, several North African countries report transplantation activity which are not reported to these registries (e.g., Egypt, Morocco, Algeria) [24]. Uzbekistan also has no captured registry data but published literature confirms transplantation activity driven predominantly with living organ donors [25]. By relying upon volunteered data registries only, organ donation and transplantation activity in Muslim-majority countries will be under-reported in our analysis but is unlikely to make any material difference to the observation of a significant shortfall in transplantation activity to meet organ failure requirements. However, this reinforces the importance of robust data capture within national or regional registries to ensure complete organ donation and transplantation activity. Not only will this allow adequate governance and oversight for healthcare provides but will provide data to determine numbers of illegal transplant tourism and/or trafficking activity. Bridging the gap between supply versus demand for organs is critical to mitigate the risk from organ trade and trafficking. People living in Muslim-majority countries will be particularly susceptible due to reported sub-optimal organ donation rates. Due to these inequities and inequalities, some may risk their health out of desperation for transplantation and contribute to the exploitation of vulnerable donors. Despite the published framework from the Declaration of Istanbul setting out country requirements for the ethical donation and transplantation of organs [26], organ trafficking or human trafficking for the purpose of organ removal remains a global challenge. A high degree of organization is needed to execute such illegal transplants, with the trade embedding transplant professionals with brokers and hospitality sectors. Healthcare professionals may directly or indirectly perpetuate illegal organ transplantation with their activity and/or complicity [27]. Underlying reasons include lack of awareness, a paucity of undergraduate and postgraduate education on organ trafficking, many simply turning a blind eye and/or the lure of significant monetary gain. There is no robust international registry to provide accurate metrics of organ trafficking or trafficking in persons for the purpose of organ removal. However, what information is available to review from data collected by the United Nations Office on Drugs and Crime suggests countries in sub-Sahara Africa, south Asia and the Gulf countries (which contain most of the Muslim-majority countries) are particularly susceptible [28, 29]. Despite prohibitive legislation, illegal transplants have been reported in Muslim-majority countries such as (but not limited to) Pakistan, Egypt and Bangladesh [30, 31]. The most effective intervention to mitigate risk of illegal and unethical transplantation is for government accountability and action to achieve national self-sufficiency in organ donation and transplantation [32].

It is important that Muslim-majority countries develop organ donation systems and transplantation infrastructure that are compatible with their strengths and abilities. For example, the countries with the highest living and deceased donor activity in our study, Turkey and Iran respectively, rank high in the world economic rankings but have evolved different organ donation and transplantation practices. Turkey performed its first live donor kidney transplant in 1975, followed by the first deceased donor kidney and liver procedures in 1978 and 1988 respectively [33]. However, their more contemporary GODT data suggests that even in Turkey the transplant program is predominantly driven by living donor activity. In contrast, the first living donor kidney transplant in Iran was performed in 1967 (living donor kidney procedure), but there was a long lag period until the first deceased donor in 2000 after legislative changes sanctioned donation after brain death [34]. Since then, Iran has made significant progress over the last two decades to build an infrastructure to promote deceased donation from brain death donors [35]. This is parallel to implementation of a novel state-regulated paid living-unrelated donor kidney transplant program in 1988 [36]. The focus on deceased donor activity (deceased organ donation rates have increased 19-fold from 2003 to 2015) has resulted in liver, pancreas, heart, and lung transplantation programs also starting in Iran and more kidney transplants are currently from deceased donors rather than living [35]. These contrasting experiences have important implications for Muslim-majority countries which may have living organ donor activity but fledgling deceased organ donor models. As per our data, deceased donation activity lags living donor activity. The example from Iran demonstrates the importance of government support, religious scholarly approval and socio-cultural confidence for establishing a national organ procurement infrastructure to maximize use of deceased organ donors. While some countries like Saudi Arabia have established such foundations [37], with increasing deceased organ donation as a result, other countries like Pakistan are lagging behind due to the lack of a collaborative mandate to establish an effective national deceased organ procurement system [38]. Investment is mandatory to support staffing, resource and infrastructure for critical services facilitating organ procurement, allocation, distribution and monitoring. While this is likely to be a significant financial undertaking, the direct and indirect cost savings are likely to be significant [39]. Stakeholder engagement to establish country-specific protocols and guidelines is vital to encourage good clinical practice and the development of efficient transplant services. This is particularly true for the development of deceased donor models. While this has the best potential to expand the donor pool, further work is necessary to encourage wider societal acceptance. Innovative methods incorporating financial incentives have been used in some Muslim-majority countries. For example, Iran can justifiably claim some success with its experience of its state-regulated, paid, unrelated living kidney donors [36]. Saudi Arabia has also nurtured its fledgling deceased donor program with monetary compensation to the deceased donor’s family (50,000 riyals) and 50% discount for travel on Saudi Airlines [19]. Columb et al. have argued for a re-conceptualization of organ commercialisation, with an important distinction to be made between “trade” and “trafficking” [40]. Finally, education for both healthcare professionals and the wider public through various platforms is key to normalize organ donation consideration as a routine aspect of end-of-life care.

The strength of our analysis is the inclusion of Muslim-majority countries from across the globe for an international perspective. Including socio-economic variables allows a more probing analysis of factors that have an association with deceased or living donor activity. Limitations of our study include that data may not be totally accurate. There was significant missing data in both GODT and IRoDaT registries regarding organ donation and transplantation activity. While for some countries it likely reflects the absence of any official transplant practice, for other countries no data was available despite published evidence of transplantation activity (e.g., Indonesia, Egypt). Correlation analyses do not imply causation and just because two variables move together it does not imply one causes the other. We did not explore the political, cultural, and/or social environment of each Muslim-majority country which are likely to be significant confounding factors. For example, factors like religious observance, the role of Islam in governance, and the diversity of Muslim practices (e.g., Sunni versus Shia belief) are influential variables in the socio-economic and cultural development of Muslim-majority countries but exploring this was beyond the scope of this work.

To conclude, organ donation and transplantation activity across Muslim-majority countries fall short of critical requirements in view of the burden of end-stage organ failure. While socio-economic factors are important, multi-factorial barriers that prevent establishment of comprehensive organ donation and transplantation services must be overcome. Development of organ donation and transplantation services, either country or region-specific, should be strongly encouraged to ensure equity of access to transplantation for people living with end-stage organ failure across global Muslim-majority countries.

## Data Availability

Publicly available datasets were analyzed in this study. This data can be found here: https://www.transplant-observatory.org; https://www.irodat.org/.

## Abbreviations

GODT, Global Observatory for Donation and Transplantation; IRoDaT, International Registry on Organ Donation and Transplantation; WHO, World Health Organization; IMF, International Monetary Fund; CKD, chronic kidney disease; GDP, Gross Domestic Product; RTA, road traffic accidents; DBD, donation after brain death; DCD, donation after circulatory death.
