# Supplementary material for: Evolving Trends in Organ Donation and Transplantation Rates Across Muslim Majority Countries
Source: Transpl Int. 2025 Nov 24;38:15116. doi: 10.3389/ti.2025.15116 (PMC12684439; doi:10.3389/ti.2025.15116)
Supplement: Supplementary file 1 [file Table1.pdf]

Supplementary Table 1. Chronic kidney disease (CKD) prevalence and deaths from the Global Burden of Disease Study

COUNTRY	CKD prevalence	Age standardised prevalence rate per 100,000	CKD deaths	Age standardised death rate per 100,00
Afghanistan	1586966.0	10913.0	3895.0	34.1
Albania	272017.0	7259.0	363.0	9.2
Algeria	3497207.0	9813.0	4577.0	15.6
Azerbaijan	1101700.0	10937.0	1169.0	14.2
Bahrain	132052.0	10427.0	133.0	22.3
Bangladesh	11196657.0	8300.0	16783.0	15.4
Bosnia and Herzegovina	427229.0	8273.0	604.0	10.5
Brunei Darussalam	40907.0	10323.0	70.0	26.0
Burkina Faso	1281101.0	11354.0	2769.0	31.9
Chad	779014.0	10861.0	1568.0	25.0
Comoros	51396.0	9900.0	104.0	26.3
Djibouti	73613.0	9851.0	139.0	30.8
Egypt	7101539.0	10572.0	13115.0	29.0
Eritrea	304308.0	9868.0	641.0	30.4
Gambia	135988.0	11336.0	243.0	25.1
Guinea	747095.0	11218.0	1598.0	28.1
Indonesia	27232922.0	11164.0	35446.0	17.3
Iran (Islamic Republic of)	8339849.0	10924.0	10163.0	16.6
Iraq	3044399.0	10991.0	4706.0	21.9
Jordan	745402.0	10378.0	1281.0	27.5
Kazakhstan	1774266.0	10093.0	1485.0	9.3
Kosovo	No data	No data	No data	No data
Kuwait	339577.0	9716.0	177.0	8.3
Kyrgyzstan	482340.0	9482.0	451.0	9.1
Lebanon	668003.0	10029.0	594.0	11.2
Libyan Arab Jamahiriya	594010.0	10963.0	1181.0	29.6
Malaysia	3187367.0	11079.0	4731.0	21.1
Maldives	41258.0	10021.0	68.0	25.5
Mali	1127953.0	10783.0	2144.0	22.7
Mauritania	271447.0	11281.0	460.0	23.8
Morocco	3289444.0	9924.0	4544.0	16.3
Niger	1047090.0	10774.0	1777.0	21.8
Nigeria	12681837.0	11387.0	13740.0	15.0
Oman	324395.0	10611.0	253.0	16.4
Pakistan	14663997.0	10162.0	36844.0	33.9

Palestine	307284.0	10423.0	624.0	28.5
Qatar	188200.0	9920.0	115.0	26.5
Saudi Arabia	2387872.0	9892.0	3818.0	29.9
Senegal	996298.0	11236.0	1930.0	27.0
Sierra Leone	510314.0	11412.0	896.0	24.1
Somalia	842140.0	10019.0	2023.0	36.0
Sudan	2274010.0	10107.0	3175.0	17.6
Syrian Arab Republic	1384897.0	9881.0	2257.0	19.7
Tajikistan	656378.0	10093.0	641.0	11.2
Tunisia	1218223.0	9916.0	1645.0	15.3
Türkiye	9042506.0	10311.0	15153.0	17.8
Turkmenistan	473830.0	10949.0	632.0	16.0
United Arab Emirates	724351.0	9951.0	829.0	30.8
Uzbekistan	2896741.0	10993.0	3584.0	15.9
Yemen	1560862.0	9680.0	1964.0	16.4
